# Dataset on chemotherapy-induced nausea and vomiting (CINV) and quality of life (QOL) during multiple chemotherapy cycles among a Chinese breast cancer patient population who were randomized to antiemetic regimens with or without olanzapine

**DOI:** 10.1016/j.dib.2020.105421

**Published:** 2020-03-12

**Authors:** Winnie Yeo, Vicky T.C. Chan, Leung Li, Thomas K.H. Lau, Kwai Tung Lai, Elizabeth Pang, Maggie Cheung, Frankie K.F. Mo

**Affiliations:** a Department of Clinical Oncology, Prince of Wales Hospital, Faculty of Medicine, The Chinese University of Hong Kong, Hong Kong SAR; b Hong Kong Cancer Institute, State Key Laboratory of Translational Oncology, The Chinese University of Hong Kong, Hong Kong SAR

**Keywords:** Cytotoxic treatment, Multiple cycles, Doxorubicin, Adriamycin, Cyclophosphamide, Quality of life (QOL), Chemotherapy-induced nausea and vomiting (CINV), Asians

## Abstract

Chemotherapy-induced nausea and vomiting (CINV) are highly distressing symptoms for cancer patients undergoing cytotoxic chemotherapy. This dataset was obtained from a homogenous group of Chinese breast cancer patients who were uniformly planned to receive a highly emetogenic (neo)adjuvant chemotherapy regimen, consisting of doxorubicin and cyclophosphamide (commonly known as AC). Patients were being randomized to one of the two antiemetic regimens: aprepitant, ondansetron and dexamethasone with (the Olanzapine arm) or without olanzapine (the Standard arm). Patients underwent self-reported diaries and questionnaires to record their nausea and vomiting symptoms, use of rescue medication as well as their quality of life (QOL). The primary and secondary endpoints have focused on efficacy analysis during the first cycle of AC chemotherapy; the results have been reported in The Breast [1]. In this Data in Brief article, we provide outcome of the analysis of data collected during multiple cycles of chemotherapy. The data reported here include the proportion of patients with “Complete Response”, “Complete Protection” and “Total Control” of emesis in the acute (0–24 h), delayed (24–120 h) and overall periods (0–120 h), as well as QOL data during all the 4 cycles of AC.

Specifications table

**Table:** 

Subject	Oncology;
Specific subject area	Chemotherapy
Type of data	Tables and Functional Living Index- Emesis (FLIE) Questionnaire [https://eprovide.mapi-trust.org/instruments/functional-living-index-emesis#need_this_questionnaire]
How data were acquired	Patients’ diaries and questionnaires to capture incidence and severity of CINV and use of rescue medication, Functional Living Index- Emesis (FLIE) questionnaire to capture patients’ QOL during chemotherapy. The diaries and questionnaires were filled in by individual patient at a university affiliated hospital and at their homes.
Data format	Raw
	Analyzed
	Filtered
Parameters for data collection	All patients were planned for 4 cycles of AC chemotherapy. Prior to each cycle of chemotherapy on Day 1, individual patient filled in self-administered FLIE questionnaire. A diary was given to each patient to bring home, so that she could record the date and time of any vomiting episodes and the use of rescue medication following the chemotherapy infusion for 120 h. Within the diary, there were also nausea ratings (by visual analogue scale, VAS; 0 mm implied no nausea; 100 mm implied nausea that was “as bad as it could be”); on days 2 to 6, each patient rated the symptoms of nausea for the preceding 24 h using the VAS. After patients had completed the diary in the morning of day 6, they immediately completed the FLIE questionnaire again.
Description of data collection	Prospective cohort of 120 adult women with breast cancer who were randomly assigned to one of the two antiemetic regimens prior to their chemotherapy. They were asked to fill in self-reported diaries and Functional Living Index- Emesis (FLIE) Questionnaire [https://eprovide.mapi-trust.org/instruments/functional-living-index-emesis#need_this_questionnaire]
Data source location	Institution: Comprehensive Cancer Trials Unit, Department of Clinical Oncology, Faculty of Medicine, The Chinese University of Hong Kong
	City/Town/Region: Hong Kong
	Country: China
Data accessibility	With the article
Related research article	Author's name: Winnie Yeo, Thomas KH Lau, Leung Li, Kwai Tung Lai, Elizabeth Pang, Maggie Cheung, Vicky TC Chan, Ashley Wong, Winnie MT Soo, Vanessa TY Yeung, Teresa Tse, Daisy CM Lam, Eva WM Yeung, Kim PK Ng, Nelson LS Tang, Macy Tong, Joyce JS Suen, Frankie KF Mo.
	Title: A Randomized Study of Olanzapine-containing versus Standard Antiemetic Regimens for the Prevention of Chemotherapy-Induced Nausea and Vomiting in Chinese breast cancer patients
	Journal: The Breast
	DOI: 10.1016/j.breast.2020.01.005.

## Value of the data

• These data over multiple cycles of chemotherapy are important for understanding and interpretations of the potential benefits of new antiemetic agents in the control of CINV [1].
• Clinicians and researchers working in the fields of oncology, palliative care and general practice may benefit from these data.
• The full description of the results provides deeper insights regarding efficacy of different antiemetic regimens when they are administered to cancer patients undergoing highly emetogenic chemotherapy.
• While data during the first chemotherapy cycle are often the primary endpoints and are thus invariably reported, data on subsequent chemotherapy cycles are rarely reported in the literature. These data provide a unique opportunity to follow symptoms and QOL of women with breast cancer who underwent 4 cycles of highly emetogenic AC chemotherapy.

## 1 Data description

In this Data in Brief article, we first provide the comparison of Complete Response (Table 1a and b), Complete Protection (Table 2a and b) and Total Control (Table 3a and b) of CINV during the acute period, delayed period and overall period across the 4 cycles of chemotherapy among patients who were randomized to one of the two study arms. The raw data based on which analyses were conducted for Complete Response, Complete Protection and Total Control are available in Supplementary File S1, Supplementary File S2 and Supplementary File S3 respectively.

**Table 1:** Proportion of patents with Complete Response (CR) during the acute period, delayed and overall time frames across the 4 cycles.

Comparison	Overall Time frame			Acute			Delayed		
	CR rate	CR rate	*p*-value	CR rate	CR rate	*p*-value	CR rate	CR rate	*p*-value
a) Olanzapine arm									
C1 vs C2	65.0	70.2	0.5502	70.0	79.0	0.2679	92.9	88.9	0.5221
C2 vs C3	70.2	75.0	0.5655	79.0	82.1	0.6680	88.9	91.3	0.6996
C3 vs C4	75.0	73.7	0.8728	82.1	82.5	0.9652	91.3	89.4	0.7514
C1 vs C3	65.0	75.0	0.2410	70.0	82.1	0.1267	92.9	91.3	0.7880
C1 vs C4	65.0	73.7	0.3090	70.0	82.5	0.1145	92.9	89.4	0.5649
C2 vs C4	70.2	73.7	0.6768	79.0	82.5	0.6350	88.9	89.4	0.9419
b) Standard arm									
C1 vs C2	38.3	57.6	0.0352	51.7	66.1	0.1097	74.2	87.2	0.1652
C2 vs C3	57.6	57.6	1.0000	66.1	66.1	1.0000	87.2	87.2	1.0000
C3 vs C4	57.6	57.6	1.0000	66.1	71.2	0.5517	87.2	81.0	0.4455
C1 vs C3	38.3	57.6	0.0352	51.7	66.1	0.1097	74.2	87.2	0.1652
C1 vs C4	38.3	57.6	0.0352	51.7	71.2	0.0288	74.2	81.0	0.4902
C2 vs C4	57.6	57.6	1.0000	66.1	71.2	0.5517	87.2	81.0	0.4455

**Table 2:** Proportion of patents with Complete Protection (CP) during the acute period, delayed period and overall time frames across the 4 cycles.

Comparison	Overall Time frame			Acute			Delayed		
	CP rate	CP rate	*p*-value	CP rate	CP rate	*p*-value	CP rate	CR rate	*p*-value
a) Olanzapine arm									
C1 vs C2	61.7	64.9	0.7159	70.0	73.7	0.6580	88.1	88.1	1.0000
C2 vs C3	64.9	67.9	0.7404	73.7	76.8	0.7026	88.1	88.4	0.9684
C3 vs C4	67.9	68.4	0.9487	76.8	77.2	0.9590	88.4	88.6	0.9692
C1 vs C3	61.7	67.9	0.4858	70.0	76.8	0.4092	88.1	88.4	0.9684
C1 vs C4	61.7	68.4	0.4440	70.0	77.2	0.3782	88.1	88.6	0.9376
C2 vs C4	64.9	68.4	0.6911	73.7	77.2	0.6634	88.1	88.6	0.9376
b) Standard arm									
C1 vs C2	36.7	55.9	0.0351	50.0	64.1	0.1123	73.3	86.8	0.1595
C2 vs C3	55.9	54.2	0.8532	64.1	64.1	1.0000	86.8	84.2	0.7444
C3 vs C4	54.2	52.5	0.8536	64.1	66.1	0.8467	84.2	79.5	0.5911
C1 vs C3	36.7	54.2	0.0542	50.0	64.1	0.1123	73.3	84.2	0.2707
C1 vs C4	36.7	52.5	0.0815	50.0	66.1	0.0752	73.3	79.5	0.5482
C2 vs C4	55.9	52.5	0.7117	64.1	66.1	0.8467	86.8	79.5	0.3890

**Table 3:** Proportion of patents with Total Control (TC) during the acute period, delayed period and overall time frames across the 4 cycles.

Comparison	Overall Time frame			Acute			Delayed		
	TC rate	TC rate	*p*-value	TC rate	TC rate	*p*-value	TC rate	TC rate	*p*-value
a) Olanzapine arm									
C1 vs C2	51.7	50.9	0.9319	65.0	59.6	0.5504	79.5	85.3	0.5177
C2 vs C3	50.9	55.4	0.6333	59.6	66.1	0.4800	85.3	83.8	0.8605
C3 vs C4	55.4	54.4	0.9174	66.1	63.2	0.7461	83.8	86.1	0.7811
C1 vs C3	51.7	55.4	0.6905	65.0	66.1	0.9034	79.5	83.8	0.6291
C1 vs C4	51.7	54.4	0.7683	65.0	63.2	0.8355	79.5	86.1	0.4490
C2 vs C4	50.9	54.4	0.7075	59.6	63.2	0.7004	85.3	86.1	0.9222
b) Standard arm									
C1 vs C2	26.7	45.8	0.0302	41.7	57.6	0.0817	64.0	79.4	0.1882
C2 vs C3	45.8	45.8	1.0000	57.6	57.6	1.0000	79.4	79.4	1.0000
C3 vs C4	45.8	47.5	0.8536	57.6	59.3	0.8518	79.4	80.0	0.9516
C1 vs C3	26.7	45.8	0.0302	41.7	57.6	0.0817	64.0	79.4	0.1882
C1 vs C4	26.7	47.5	0.0188	41.7	59.3	0.0541	64.0	80.0	0.1671
C2 vs C4	45.8	47.5	0.8536	57.6	59.3	0.8518	79.4	80.0	0.9516

In summary, there was no difference on Complete Response, Complete Protection and Total Control across the four cycles in the Olanzapine arm. However, in the Standard arm, some differences were observed in Complete Response, Complete Protection and Total Control when comparing cycle 1 with other cycles.

We also provide data on QOL data in the overall period during the 4 cycles of chemotherapy in the two study arms. The raw data are available in Supplementary File S4. Based on the data, differences were only detected between the 2 study arms in cycle 1.

## 2 Experimental design, materials, and methods

The original study consisted of a homogenous group of Chinese breast cancer patients who were uniformly planned to receive (neo)adjuvant AC chemotherapy. Patients were being randomized to one of the two antiemetic regimens: aprepitant, ondansetron and dexamethasone with or without olanzapine.

Between the two study arms, the primary objective was to compare the antiemetic efficacies in the first cycle of AC. The secondary objectives were to compare QOL in the first cycle of AC and to compare the tolerability and efficacy of the study treatments.

All patients were planned for 4 cycles of AC chemotherapy. Data collected included patients’ baseline characteristics. In addition to cycle 1, patients’ symptoms and QOL were captured during 2nd–4th cycle of chemotherapy. Individual patient filled in self-administered FLIE questionnaire on day 1 before AC chemotherapy. After chemotherapy, the patients went home with the provision of a diary. Each patient recorded the date and time of any vomiting episodes and the use of rescue medication in the first 120 h after AC chemotherapy. On days 2 to 6, each patient rated the symptoms of nausea for the preceding 24 h using the VAS in the diary. After patients had completed the diary in the morning of day 6, they immediately completed the FLIE questionnaire again.

The primary and secondary study outcomes related to outcomes in cycle 1 have been reported [1]. In this Data in Brief article, we provide outcome of the analysis of data collected during multiple cycles of AC. Three time-frames were assessed during each AC chemotherapy cycle; assessments started from the initiation of AC chemotherapy infusion (0 h) up to beginning of day 6 (∼120 h). “Acute” period referred to 0–24 h after the initiation of AC, “delayed” period referred to 24–120 h, while “overall” period referred to 0–120 h The variables measured were the proportion of patients with “Complete Response”, “Complete Protection” and “Total Control”, definitions of which have been provided in the original report [1] as well as previous studies [2], [3], [4]. These assessments were done primarily over the “overall” period, and were also conducted separately during “acute” and “delayed” periods of each chemotherapy cycle. QOL data was collected during the overall period of each chemotherapy cycle.

For efficacy analyses over multiple cycles, “Complete Response”, “Complete Protection”, and “Total Control” over multiple cycles in the acute (0–24 h), delayed (24–120 h) and overall periods (0–120 h) were assessed using chi-square test for dichotomous data. For the analysis of the FLIE questionnaire, the nausea domain, vomiting domain and total score (the sum of the two domains) in the overall period were compared between the two arms using Wilcoxon Rank Sum test for continuous data.

## Appendix Supplementary materials

Image, application 1

Image, application 2

Image, application 3

Image, application 4
